# Cardiovascular Risk Distribution in a Contemporary Polish Collective

**DOI:** 10.3390/ijerph17093306

**Published:** 2020-05-09

**Authors:** Anita Liput-Sikora, Anna Maria Cybulska, Wiesława Fabian, Anna Fabian-Danielewska, Marzanna Stanisławska, Magdalena Sylwia Kamińska, Elżbieta Grochans

**Affiliations:** 1 Primary Care Center (NZOZ Przychodnia Medycyny Rodzinnej SJ), ul. Kadłubka 10-11, 71-521 Szczecin, Poland; anita.liputsikora@gmail.com (A.L.-S.); Wifab@poczta.onet.pl (W.F.); anna1.fabian@gmail.com (A.F.-D.); 2 Department of Nursing, Pomeranian Medical University in Szczecin, Żołnierska 48, 71-210 Szczecin, Poland; stamarz@pum.edu.pl (M.S.); grochans@pum.edu.pl (E.G.); 3 Faculty of Medicine, Pomeranian Medical University in Szczecin, Rybacka 1, 70-204 Szczecin, Poland; 4 Subdepartment of Long-Term Care, Department of Social Medicine, Pomeranian Medical University in Szczecin, Żołnierska 48, 71-210 Szczecin, Poland; magdalena.kaminska@pum.edu.pl

**Keywords:** cardiovascular diseases, risk factors, prevention

## Abstract

The aim of this study was to assess the prevalence of selected risk factors for cardiovascular disease (hypertension, overweight, obesity, carbohydrate metabolism disorders, a positive family history, a lack of physical activity), and to estimate the risk of a cardiovascular incident according to the Systematic Coronary Risk Evaluation (SCORE) algorithm for patients aged 35, 40, 45, 50, and 55 years, included in a primary-care prevention program, with regard to selected variables (sex and age brackets). The study sample consisted of 2009 subjects, 63% of whom were women. The largest group was the group of 35-year-olds (27%). The research method was the analysis of medical documentation of primary-care patients living in West Pomerania included in the Program of Prevention and Early Detection of Cardiovascular Disease of the National Health Fund. We collected data concerning risk factors for cardiovascular disease, blood pressure, anthropometric measurements (arm circumference, waist circumference, height, weight), body mass index (BMI), and the levels of total cholesterol (TC), low-density lipoprotein cholesterol (LDL-C), high-density lipoprotein cholesterol (HDL-C), triglycerides (TG), and fasting glucose, as well as the SCORE results. Men more often than women were overweight and obese, had hyperglycemia, and had elevated levels of total cholesterol, LDL cholesterol, and triglycerides (*p* < 0.001). There was also a statistically significant difference in the odds of a cardiovascular incident (*p* < 0.001)—the SCORE results obtained by men were higher. Men require special preventive measures in order to reduce their risk factors for cardiovascular disease, especially hypertension, dyslipidemia, diabetes, overweight, obesity, smoking, and a positive family history.

## 1. Introduction

Cardiovascular disease (CVD) is a class of social diseases that are the leading cause of death on a global scale; 17.9 million people die from cardiovascular diseases each year, with four out of five deaths due to strokes and myocardial infarctions [1,2]. In Poland, cardiovascular disease is the most common cause of death for men over 45 and women over 70 years of age. Women die from cardiovascular disease more often than men, which, however, results from their older age structure. If the differences in age structures between both sexes are eliminated, it becomes clear that cardiovascular disease poses a greater threat to the lives of men. Recent data show that men’s mortality rate in Poland is higher by 109% and women’s by 87% compared with the mean mortality rate in other countries of the European Union. What is more, the average life expectancy in Poland is 3–7 years shorter than in other 15 European Union countries [2]. 

The INTERHEART analysis revealed that about 90% of myocardial infarction cases in men and 94% of such cases in women can be attributed to nine modifiable risk factors [3]. The IMPACT project, carried out in Poland, demonstrated that treatment for acute episodes of ischemic heart disease reduced a mortality rate only by about 9%, while change in a lifestyle resulted in a 52% decline in the death rate for men and a 60% decline for women [3,4]. 

There are many classifications of risk factors for cardiovascular disease. One of the most important is the division into modifiable and non-modifiable risk factors. The first group includes hypertension, elevated total cholesterol (TC) and low-density lipoprotein cholesterol (LDL-C) levels, a low high-density lipoprotein cholesterol (HDL-C) level, diabetes, smoking, obesity, a lack of physical activity, bad diet, as well as inflammatory factors and post-thrombotic syndrome. Non-modifiable risk factors are age, sex, a family history of early cardiovascular disease, a positive history of cardiovascular disease, and genetic factors [5,6]. Another important contributor to the pathobiology of atherosclerosis and cardiovascular disease is a dysfunction of the endothelial lining of lesion-prone areas of the arterial vasculature [7].

As indicated by a high mortality rate from this condition, cardiovascular disease is a serious problem in Poland. Despite preventive actions addressed to people aged 35, 40, 45, 50, and 55 years, it is the main cause of death for Poles. Prevention of cardiovascular disease in primary healthcare is directed to individuals without a diagnosis of atherosclerotic cardiovascular disease or diabetes. 

The aim of this study was to assess the prevalence of selected risk factors for cardiovascular disease (hypertension, overweight, obesity, carbohydrate metabolism disorders, a positive family history, a lack of physical activity), and to estimate the risk of a cardiovascular incident ending in death within 10 years according to the Systematic Coronary Risk Evaluation (SCORE) algorithm for patients aged 35, 40, 45, 50, and 55 years, included in a primary care prevention program with regard to sex and age brackets (35–44 vs. 44–55 years). 

## 2. Materials and Methods 

### 2.1. Study Group

The research method was analysis of the medical record documentation of patients included in the Cardiovascular Disease Prevention Program in the years 2012, 2013, 2017, and 2018. 

The study sample consisted of 2009 patients of the outpatient clinic in Szczecin, Westpomeranian Province. 

The inclusion criteria were:

- place of residence in Westpomeranian Province;
- participation in the Cardiovascular Disease Prevention Program financed by the National Health Fund in the years 2012, 2013, 2017, and 2018;
- age of 35, 40, 45, 50, 55 years;

The exclusion criteria were:

- early diagnosis of cardiovascular diseases;
- using services provided as part of the Cardiovascular Disease Prevention Program during the previous five years.

Patients were recruited through invitation letters and paper flyers in the outpatient clinic. The study was carried out using a cardiovascular disease screening card in accordance with the instruction published by the National Health Fund. The research was approved by the Bioethical Commission of the Pomeranian Medical University, Szczecin on 19 November 2018. 

### 2.2. Procedure

The study involved several stages. First, we collected general data concerning the patients (age, sex) and their risk factors for cardiovascular disease (a positive family history, low physical activity, smoking, hypertension). Next, we took anthropometric measurements, such as weight [kg] and height [cm], rounded to the nearest 0.1 kg and 0.1 cm, respectively. We used an approved medical scale with an integrated measuring rod (SECA 711), and followed the standard procedure. Additionally, we measured arm [cm] and waist circumference [cm] for each patient. At the second stage, blood pressure was measured twice using a sphygmomanometer. The patients were sitting with their arms resting on a hard surface. The values of pressure were expressed in millimeters of mercury (mmHg). 

### 2.3. Sampling Analyses

Next, we collected whole blood to Vacutainer tubes. The patients had an empty stomach (there was a 12-h break between the last meal and blood collection). Blood samples for laboratory analysis were drawn in the blood collection point between 7.30 a.m. and 10.30 a.m. The levels of total cholesterol [mg/dl], HDL cholesterol [mg/dl], LDL cholesterol [mg/dl], triglycerides (TG) [mg/dl], and fasting glucose [mg/dl] were determined. 

### 2.4. Systematic Coronary Risk Evaluation (SCORE)

At the last stage of the study, we entered the data (anthropometric measurements, blood parameters) to the form on the website of the Computer System for the Monitoring of Prevention of the National Health Fund. The program calculated body mass index (BMI) according to the formula: BMI = weight [kg] : height2 [m2]. Next, we estimated the risk of death due to acute cardiovascular incidents within 10 years according to the SCORE algorithm. Normal values accepted for the parameters and laboratory tests are shown in Table 1.

The risk of death from cardiovascular disease in 10 years was estimated according to the SCORE as follows: small <1%, moderate 1–4%, high 5–9%, and very high ≥10% [4].

### 2.5. Statistical Analysis

Statistical analysis was performed using Statistica v.13.3 (TIBCO^®^ Statistica, Palo Alto, USA). All variables were analyzed using descriptive statistics. The following parameters were determined for quantitative variables: measures of central tendency, measures of dispersion, measures of location. In the case of variables measured on a nominal or an ordinal scale, a measure of the structure (frequency) was established. Based on the central limit theorem that in large samples (N > 100) quantitative variables have a distribution similar to normal, parametric statistics were used to analyze these variables. The results obtained for the groups of women and men, as well as for age groups (35–44 vs. 44–55) were compared using Student’s t-test for independent samples. The effect size for the difference between the groups was estimated using Cohen’s d coefficient. The size of the difference was regarded as: small for (d: 0.10–0.30), average for (d: 0.31–0.50), and big for (d > 0.50) [8]. 

## 3. Results

The study sample consisted of 2009 subjects, the majority of whom (677) entered the study in 2017, and the rest (580) in 2018. Considerably more women (63%) than men (37%) came forward to take part in the study. The largest groups were the group of 35-year-olds (27% of the results obtained), and the group of 40-year-olds (24%). The smallest group was a group of 55-year-olds (14%). 

### 3.1. Analysis of the Frequency of Selected Risk Factors for Cardiovascular Disease 

The data concerning the incidence of selected risk factors for cardiovascular disease in the studied population revealed that almost half (49.5%) of the respondents had normal weight, 35.2% were overweight, and about 14% were obese. Hypertension was noted in more than 7% of the patients. Detailed analysis of the data showed that 7% of the respondents had systolic blood pressure above normal, and 7.3% had elevated diastolic blood pressure. 

Among the respondents 40.9% had normal total cholesterol levels; 50.1% had increased serum levels of LDL cholesterol. Normal HDL cholesterol levels were observed in 92.6% of the patients. Only 7.3% of the participants had levels of this parameter below normal. 84.9% of the participants had normal levels of triglycerides. The results showed that 11.9% of the population had impaired fasting glycemia, and nine participants (0.4% of the study sample) had diabetes.

Over half of the participants (53.4%) claimed that they had never smoked, about one-fifth (23.3%) stated that they had smoked in the past, and 23.3% reported that they currently smoked. Furthermore, 73.2% of the participants did not have the minimum recommended physical activity (>3 times a week). The moderate physical activity group (3–5 times a week) included slightly more than one-fifth of the respondents (20.6%), and only 6.2% had physical effort more than five times a week.

A vast majority of the respondents (88.9%) claimed that their mothers had stroke and/or a myocardial infarction before the age of 60 years, and fathers before the age of 55 years. About 11% of the respondents had a positive family history for one parent only, and 0.2% had a positive family history for both parents. 

The data indicate that almost half of the studied primary-care patients had a small risk (<1%) of a cardiovascular incident ended in death in ten years, 46.8% had a moderate risk (1%–4%), and 4.2% had either a high (5%–9%) or a very high risk (≥10%). 

### 3.2. Analysis of the Prevalence of Selected Risk Factors for Cardiovascular Disease with Regard to Sex

Analysis revealed statistically significant relationships between selected risk factors for cardiovascular disease and sex. Women had considerably more often a positive family history (*p* = 0.045). Another statistically significant parameter (*p* < 0.001) was smoking―more men than women (28.5% vs. 20.2%) were smokers at the moment of the study, and more men smoked in the past (29.2% vs. 19.8%). A statistically significant parameter (*p* = 0.048) was also physical effort―men were more keen to undertake physical effort than women. Furthermore, statistically significant differences (*p* < 0.001) between sexes were observed for systolic blood pressure―the odds in favor of increased systolic blood pressure were twice those for men than for women (odds ratio (OR) = 3.17). A statistically significant difference (*p* < 0.001) was found in body mass index (BMI) values―overweight and obese men were more numerous than women. Laboratory analysis demonstrated statistically significant differences (*p* < 0.001) in the levels of glucose, total cholesterol, LDL cholesterol, HDL cholesterol, triglycerides―men more often had hyperglycemia and elevated levels of total cholesterol, LDL cholesterol, and triglycerides. The odds in favor of increased triglyceride levels were over four times higher (OR = 4.32), and the odds of decreased HDL cholesterol levels were twice those (OR = 2.00) for men. There was also a statistically significant difference in the odds of a cardiovascular incident (*p* < 0.001)―the SCORE results obtained by men were higher (Table 2). 

The results for all variables―except for age and pulse―were statistically significant (*p* < 0.001). In the case of other variables, the mean results obtained by men were higher than those achieved by women―the only exception was the level of HDL cholesterol (higher in women). A parameter that sex had the strongest effect on was waist circumference (d = 1.162) (Table 3). 

### 3.3. Analysis of the Prevalence of Selected Risk Factors for Cardiovascular Disease with Regard to Age Brackets 

Analysis of the relationship between selected parameters and an age bracket demonstrated statistically significant differences in smoking, blood pressure, glycemia, BMI, total cholesterol, LDL cholesterol, triglycerides, and the SCORE results. 

The group of 45- to 55-year-olds included a higher percentage of smokers and more people who had given up smoking, while in the younger age group there were substantially more individuals who had never smoked. Systolic blood pressure was significantly higher in the older age bracket, and the odds of increased values were over twice higher than in the younger age bracket. Diastolic blood pressure was significantly lower in the 45- to 55-year-olds. The odds of increased diastolic blood pressure were 38% lower in the older age bracket. The percentage of overweight and obese patients was higher in the older age bracket. A similar observation was made for blood parameters i.e., glucose, total cholesterol, LDL cholesterol, and triglycerides. The percentage of people with increased values of these parameters was significantly higher in the 45–55 years age group than in the group of 35- to 44-year-olds; the odds of elevated triglyceride levels were 34% higher in the age group of 45–55 years than in the age group of 35–44 years. The percentage of the participants with high SCORE results in the 45–55 years age group was considerably higher than in the group of 35- to 44-year-olds (Table 4).

The mean values of waist circumference, BMI, systolic RR, diastolic RR, glycemia, total cholesterol (the strongest effect: d = 0.484), LDL cholesterol, and triglycerides were statistically significantly lower (*p* < 0.005) in the 35–44 years age group compared with the group of 45- to 55-year-olds (Table 5).

## 4. Discussion

The studied West Pomeranian population was burdened with many risk factors for cardiovascular disease: hypertension, dyslipidemia, diabetes, overweight and obesity, low physical activity, smoking and a family history of early cardiovascular disease. Men and the elderly were characterized by a greater severity of these factors. 

A review of the literature indicates that scientists are concentrating on specific risk factors for cardiovascular disease. In the discussion we focused on three of four: diabetes, elevated blood pressure, dyslipidemia, and obesity.

As stated by Wild et al., over 170 million people worldwide suffer from diabetes―one of the main risk factors for cardiovascular disease, which is the most common cause of death in this group [9]. Data from the National Institute of Public Health show that in Poland more than 2.5 million people suffer from diabetes [10]. In our study, people with diabetes accounted for 0.4% of the study sample, whereas those with pre-diabetes constituted 11.5%. Diabetes and pre-diabetes were significantly more often observed in men and in the older age group (45–55 years). Nationwide analysis covering the years 2008, 2010, 2013, 2014 demonstrated that the percentage of diabetic men decreased to 3.5%, 2.9%, 1.9%, respectively, up to 2014 when it increased to 2.1%. In the study conducted by Kwaśniewska and Drygas, in 2014, pre-diabetes was noted in 10.7% of women and 19.6% of men [2]. In 2005, Fabian et al. observed type 2 diabetes in 3.59% of primary-care patients in West Pomerania. The percentages of diabetic patients in the groups of women and men were slightly different (3.9% vs. 3.11%), and were significantly higher than in our research. As in our study, diabetes was more prevalent in older age groups [11].

The EUROASPIRE III and EUROASPIRE IV (European Action on Secondary Prevention by Intervention to Reduce Events) survey showed that hypertension is the main cardiovascular risk factor [12,13]. 

As confirmed by the American epidemiological research, hypertension is a cause of 40.6% of deaths from cardiovascular disease [14]. In our study, hypertension affected 7% of the population analyzed, including 4.1% of women and 12% of men. According to the World Health Organization (WHO) report from 2015, such differences in the incidence of hypertension among men and women are also observed in other countries with a similar socioeconomic profile, among them Lithuania (35.3% vs. 24.3%), the Czech Republic (33.2% vs. 21%), and Hungary (34.6% vs. 23.1%). A similar, although less strong, trend was observed in Western Europe, for example England (23.3% vs. 20.7%) and Germany (25.1% vs. 22.9%) [15]. 

Another risk factor for cardiovascular disease is dyslipidemia. Increased lipid values are often related to other risk factors (bad eating habits, a lack of physical effort, smoking) which, when uncontrolled, worsen the course of the disease. People with lipid profile disorders are at twice the risk of cardiovascular disease than those without dyslipidemia. As Karr asserts, more than 100 million US citizens (about 53% of the adult population) have elevated LDL cholesterol levels [16,17]. The first study conducted to assess the incidence of dyslipidemia in Poland was the Pol-MONICA (Polish Monitoring Trends and Determinants in Cardiovascular Diseases) project performed in 1984–1993. It showed that 70% of women and 73% of men had hypercholesterolemia. Elevated LDL levels were more common in men (53% of women and 60% of men) [18]. Our analysis demonstrated increased LDL levels in 50.1% of the studied population, including 44.2% of women and 60.7% of men. 

Another factor predisposing to cardiovascular diseases is overweight. The EUROASPIRE IV survey revealed large differences between European countries in lifestyles and risk-factor management, the use of cardioprotective medication, the provision of cardiac prevention and rehabilitation, and other preventive services [12,13]. Women more often than men had obesity, abdominal overweight, and central obesity. EUROASPIRE IV demonstrated that elevated blood pressure, raised LDL-C, and self-reported diabetes mellitus were slightly more common among women than among men [13].

According to Andolfi et al., obese people currently constitute 33% of the US population, which is estimated to increase to 50% by 2030 [19]. In the Polish Multi-centre National Population Health Examination Survey WOBASZ I project (carried out in 2003–2005), overweight was detected in 40.4% of men and 27.9% of women, and obesity was observed in 20.6% of men and 20.2% of women in the population of 20- to 74-year-olds. In the NATPOL project performed in 2011 to assess the prevalence and control of cardiovascular diseases risk factors in Poland, obesity was more common in men than in women (23.6% vs. 19.7%). The mean BMI values were 27.2 kg/m2 for men and 26.1 kg/m2 for women [2]. In our investigation, obesity was observed in 14% of the participants, however the percentage of obese men was significantly higher than the percentage of obese women, which corresponds with the aforementioned findings. It was interesting that men were more keen to undertake physical effort than women. According to Lanier et al. and Orkaby et al., physical activity is an important protective factor against cardiovascular disease. However, in our study men were more often diagnosed with moderate, high, and very high risk of a cardiovascular incident ending in death than women [20,21].

Summing up, in our study nearly half of the respondents were at low risk of a cardiovascular incident leading to death in 10 years. High SCORE results were significantly more often obtained by men than by women (73.8% vs. 31% for moderate risk; 11.2% vs. 0.1% for high and very high risk). Matyjaszczyk et al. informed about a slightly higher percentage of subjects with a low risk (52%), 31% with a moderate risk, and 17% with a high risk. They analyzed similar age groups and found that 56.4% of men at the age of 46–55 years were at a high risk [22]. In our study, this group only included 7.1% of men, however as many as 67.8% had a moderate risk for a cardiovascular incident. Maciąg et al., on the other hand, noted that 5.05% had an increased risk, with ‘increased’ meaning the SCORE result >5%, which corresponds with 4.2% of the population in our study. Maciąg et al. noted a higher risk in 1.32% of women and 11.49% of men―as for men, our research confirmed this result, but the percentage of women was significantly lower [23]. 

## 5. Conclusions

- The study sample had multiple risk factors for cardiovascular disease, the most common of which were hypertension, dyslipidemia, diabetes, overweight, obesity, low physical activity, smoking, and a family history of early cardiovascular disease.
- In the studied population, men and elderly people were the groups requiring special preventive actions reducing risk factors for cardiovascular disease (especially hypertension, dyslipidemia, diabetes, overweight and obesity, smoking).
- According to the SCORE results, men were more often diagnosed with moderate, high, and very high risk of a cardiovascular incident ending in death than women.

## Figures and Tables

**Table 1:** Normal values for parameters and laboratory tests measuring the risk of cardiovascular disease.

Variables	Range
**Blood pressure**	SBP > 140 mmHg or DBP > 90 mmHg hypertension: in two independent measurements: SBP >140 mmHg and/or DBP >90 mmHg
**Body weight**	underweight < 18.5 [kg/m^2^] normal weight of 18.5–25 [kg/m^2^] overweight 25–29.9 [kg/m^2^] obesity ≥ 30 [kg/m^2^]
**Visceral obesity**	waist circumference: men > 94 cm, women > 80 cm
**Diabetes**	fasting glucose ≥ 126 [mg/dl]
**Prediabetes**	fasting glucose 100–125 [mg/dl]
**Lipid panel**	TC < 190 [mg/dl] LDL-C ≤ 115 [mg/dl] HDL-C ≥ 40 [mg/dl] for men; ≥ 45 [mg/dl] for women TG > 150 [mg/dl]
**Low physical activity**	at least 30 min < 3 times a week
**Smoking**	>1 cigarette per day
**Positive family history**	stroke and/or a myocardial infarction―father before the age of 55 years; mother before the age of 60 years

SBP-systolic blood pressure DBP-diastolic blood pressure SCORE- Systematic Coronary Risk Evaluation TG- triglycerides TC- total cholesterol LDL-C -low-density lipoprotein cholesterol HDL-C-high-density lipoprotein cholesterol.

**Table 2:** Selected parameters with regard to sex.

Variable		Women		Men		χ2	*p* *	OR (95% CI)
		n	%	n	%			
Genetics	negative	1108	87.6	678	91.1	6.187	0.045	
	1 parent	154	12.2	64	8.6			
	2 parents	3	0.2	2	0.3			
Smoking	never	758	59.9	315	42.3	58.313	0.000	
	gave up smoking	251	19.8	217	29.2			
	smokes	256	20.2	212	28.5			
Physical activity	<3 times a week	948	75.0	522	70.2	6.041	0.048	
	3–5 times a week	240	19.0	174	23.4			
	>5 times a week	76	6.0	48	6.5			
SBP [mmHg]	normal	1213	95.9	655	88.0	44.256	0.000	3.17 (2.22; 4.52)
	elevated	52	4.1	89	12.0			
DBP [mmHg]	normal	1177	93.0	685	92.1	0.654	0.418	1.15 (0.82; 1.62)
	elevated	88	7.0	59	7.9			
Body mass index	underweight	20	1.6	2	0.3	164.29	0.000	
	normal	758	60.0	237	31.9			
	overweight	344	27.2	363	48.9			
	obesity	141	11.2	141	19.0			
Glycemia [mg/dl]	normal	1164	92.0	604	81.2	52.371	0.000	
	prediabetes	98	7.7	134	18.0			
	diabetes	3	0.2	6	0.8			
TC [mg/dL]	≤190	580	45.8	241	32.6	45.859	0.000	
	191–250	602	47.6	402	54.4			
	>250	83	6.6	96	13.0			
LDL-C [mg/dL]	≤115	705	55.9	292	39.2	52.144	0.000	
	116–130	180	14.3	152	20.4			
	>130	376	29.8	300	40.3			
HDL-C [mg/dL]	normal	1193	94.5	667	89.7	16.530	0.000	2.00 (1.42; 2.80)
	decreased	69	5.5	77	10.3			
TG [mg/dL]	≤150	1164	92.0	541	72.7	135.89	0.000	4.32 (3.34; 5.61)
	>150	101	8.0	203	27.3			
SCORE	<1%	872	68.9	112	15.1	598.37	0.000	
	1%–4%	392	31.0	549	73.8			
	5%–9%	1	0.1	69	9.3			
	≥10%	0	0.0	14	1.9			

χ2—chi-square test, * test chi2 Pearsona p—statistical significance, OR—odds ratio, SBP—systolic blood pressure, DBP—diastolic blood pressure, SCORE—Systematic Coronary Risk Evaluation.

**Table 3:** Mean values of selected parameters with regard to sex.

Variable	Women		Men		t	*p* *	d (95% CI)
	M	SD	M	SD			
Age [years]	43.2	6.9	43.4	7.0	−0.760	0.447	0.035 (−0.056; 0.125)
Arm circumference [cm]	27.7	3.5	31.2	3.6	−20.967	0.000	0.972 (0.876; 1.067)
Waist circumference [cm]	81.3	11.2	94.3	11.2	−25.133	0.000	1.162 (1.064; 1.259)
BMI	24.7	4.4	26.9	3.9	−11.533	0.000	0.533 (0.441; 0.625)
SBP [mmHg]	113.4	12.2	121.8	13.2	−14.451	0.000	0.668 (0.575; 0.761)
DBP [mmHg]	72.7	7.6	76.9	8.2	−11.710	0.000	0.540 (0.448; 0.632)
Pulse [ud/min]	72.6	6.9	72.6	6.9	−0.074	0.941	0.004 (−0.086; 0.095)
Glycemia [mg/dL]	88.8	8.8	93.6	13.0	−9.838	0.000	0.454 (0.362; 0.545)
TC [mg/dL]	198.7	35.5	207.1	37.8	−4.992	0.000	0.231 (0.140; 0.321)
LDL-C [mg/dL]	111.7	31.5	124.7	34	−8.615	0.000	0.399 (0.307; 0.490)
HDL-C [mg/dL]	68.9	17.0	56.6	16.6	15.801	0.000	−0.730 (−0,823; −0.636)
TG [mg/dL]	90.7	48.9	133.6	78.7	−15.085	0.000	1.050 (0.953; 1.146)

M—mean, SD—standard deviation, d—Cohen’s coefficient (effect size), CI—confidence interval * Student’s *t*-test, *p*—statistically significance, BMI—body mass index, SBP—systolic blood pressure, DBP—diastolic blood pressure.

**Table 4:** Selected parameters with regard to age group.

Variable		35–44		45–55		χ2	*p* *	OR (95% CI)
		n	%	n	%			
Sex	women	651	63.6	614	62.3	0.331	0.565	1.5 (0.88; 1.6)
	men	373	36.4	371	37.7			
Genetics	negative	909	88.8	877	89.0	1.690	0.430	
	1 parent	111	10.8	107	10.9			
	2 parents	4	0.4	1	0.1			
Smoking	never	590	57.6	483	49.0	16.244	0.000	
	gave up smoking	226	22.1	242	24.6			
	smokes	208	20.3	260	26.4			
Physical activity	<3 times a week	756	73.8	714	72.6	5.275	0.072	
	3–5 times a week	217	21.2	197	20.0			
	>5 times a week	51	5.0	73	7.4			
SBP [mmHg]	normal	979	95.6	889	90.3	22.034	0.000	2.35 (1.63; 3.39)
	elevated	45	4.4	96	9.7			
DBP [mmHg]	normal	933	91.1	929	94.3	7.588	0.006	0.62 (0.44; 0.87)
	elevated	91	8.9	56	5.7			
Body mass index	underweight	11	1.1	11	1.1	23.955	0.000	
	normal	562	54.9	433	44.0			
	overweight	321	31.4	386	39.3			
	obesity	129	12.6	153	15.6			
Glycemia [mg/dl]	normal	939	91.7	829	84.2	27.028	0.000	
	prediabetes	82	8.0	150	15.2			
	diabetes	3	0.3	6	0.6			
TC [mg/dL]	≤190	537	52.7	284	28.8	127.40	0.000	
	191–250	428	42.0	576	58.5			
	>250	54	5.3	125	12.7			
LDL-C [mg/dL]	≤115	580	56.9	417	42.3	50.290	0.000	
	116–130	166	16.3	166	16.9			
	>130	274	26.9	402	40.8			
HDL-C [mg/dL]	normal	958	93.6	902	91.9	2.150	0.143	1.29 (0.92; 1.81)
	decreased	66	6.4	80	8.1			
TG [mg/dL]	≤150	888	86.7	817	82.9	5.570	0.018	1.34 (1.05; 1.72)
	>150	136	13.3	168	17.1			
SCORE	<1%	751	73.3	233	23.7	521.93	0.000	
	1%–4%	273	26.7	668	67.8			
	5%–9%	0	0.0	70	7.1			
	≥10%	0	0.0	14	1.4			

OR–odds ratio, 95% CI–95% confidence interval, * Pearson’s chi-square test, SBP–systolic blood pressure, DBP–diastolic blood pressure, SCORE–Systematic Coronary Risk Evaluation.

**Table 5:** The mean values of selected parameters with regard to age bracket.

Variable	35–44		45–55		t	*p* *	d (95% CI)
	M	SD	M	SD			
Arm circumference [cm]	28.9	4.1	29.1	3.7	−0.836	0.403	0.036 (–0.052; 0.123)
Waist circumference [cm]	85.2	12.8	87.1	12.8	–3.293	0.001	0.147 (0.059; 0.235)
BMI	25.1	4.5	25.9	4.2	–3.878	0.000	0.174 (0.087; 0.262)
SBP [mmHg]	114.7	11.4	115.2	12.9	–0.682	0.496	0.05 (–0.11; 0.21)
DBP [mmHg]	73	7.2	74.4	7.4	–2.802	0.005	0.19 (0.03; 0.36)
Pulse [ud/min]	72.3	6.9	72.9	6.9	–1.792	0.073	0.080 (–0.008; 0.167)
Glycemia [mg/dL]	89	10.5	92.3	10.9	–6.735	0.000	0.300 (0.213; 0.388)
TC [mg/dL]	193.4	35	210.6	36.1	–10.838	0.000	0.484 (0.395; 0.572)
LDL-C [mg/dL]	109.1	30.4	124.2	33.8	–10.493	0.000	0.469 (0.380; 0.557)
HDL-C [mg/dL]	64.1	17.8	64.6	18	–0.671	0.502	0.030 (–0.058; 0.117)
TG [mg/dL]	10	63.7	112.4	65.9	–3.933	0.000	0.176 (0.088; 0.263)

M—mean, SD—standard deviation, d—Cohen’s coefficient (effect size), * Student’s *t*-test, *p*—statistical significance, BMI—body mass index, SBP—systolic blood pressure, DBP—diastolic blood pressure.
